# 80. The Utility of Cultures for Isolated Fevers in Patients with Influenza or COVID-19 Receiving Extracorporeal Membrane Oxygenation

**DOI:** 10.1093/ofid/ofab466.282

**Published:** 2021-12-04

**Authors:** Joseph E Marcus, Valerie Sams, Michal Sobieszcyk, Alice E Barsoumian

**Affiliations:** 1 San Antonio Uniformed Services Health Education Consortium, San Antonio, Texas; 2 Brooke Army Medical Center, San Antonio, Texas; 3 SAUSHEC, San Antonio, Texas

## Abstract

**Background:**

Critically ill patients receiving extracorporeal membrane oxygenation (ECMO) are at elevated risk for nosocomial infection. Physiological responses to infection on ECMO are difficult to interpret as many clinical characteristics are controlled by the circuit including temperature. This study aimed to determine the culture positivity rates in patients receiving ECMO with influenza or COVID-19.

**Methods:**

A single center retrospective study was performed on all patients who received ECMO support at a single institution between December 2014 and December 2020 with influenza or COVID-19. All cultures ordered were reviewed for indication. Patients with fever without specific clinical syndrome or signs of decompensation, such as increasing vasopressor requirement were included. Infections and contaminants were defined by treatment team.

**Results:**

A total of 45 patients received ECMO with an admission diagnosis of influenza or COVID-19 during the study period. This cohort had a median age of 44 (interquartile range (IQR): 36-53) and was predominantly male (84%). The median time on ECMO was 360 hours (IQR: 183-666). 43/137 (31%) of infectious workups were ordered for isolated fever. The most common workup ordered for fever was combination blood cultures (BC) and urine cultures (UC) (13, 30%), followed by combination BC, UC, and respiratory cultures (RC) (11, 26%). Four (9%) infections were identified (3 blood stream, 1 respiratory) and five (12%) cultures grew contaminants (1 blood, 1 respiratory, 2 urine). Culture positivity rate was greatest for BC (3/35, 9%) followed by RC (1/19, 5%), and lowest for UC (0/26, 0%).

**Conclusion:**

Although cultures are commonly ordered for isolated fever in patients with influenza and COVID-19 receiving ECMO, culture positivity rate is low. In particular, no urinary tract infections were identified and the screening for urinary tract infection in patients receiving ECMO with isolated fever is not beneficial. Further work identifying signs and symptoms associated with infection is needed to improve diagnostic stewardship in this population that is high risk for nosocomial infections.

**Disclosures:**

**All Authors**: No reported disclosures

